# Impact of airline network on the global importation risk of mpox, 2022

**DOI:** 10.1017/S0950268823000456

**Published:** 2023-03-21

**Authors:** Ryo Kinoshita, Miho Sassa, Shogo Otake, Fumi Yoshimatsu, Shoi Shi, Ryo Ueno, Motoi Suzuki, Daisuke Yoneoka

**Affiliations:** 1 National Institute of Infectious Diseases, Tokyo, Japan; 2 Kyoto University School of Public Health, Kyoto, Japan; 3 Graduate School of Medicine, The University of Tokyo, Tokyo, Japan; 4 Department of Pediatrics, Graduate School of Medicine, Kobe University, Hyogo, Japan; 5 The Australian and New Zealand Intensive Care Research Centre, Melbourne, Australia; 6 Department of Health Policy and Management, School of Medicine, Keio University, Tokyo, Japan; 7 Tokyo Foundation for Policy Research, Tokyo, Japan

**Keywords:** Airline transportation network, effective distance, importation risk, mpox, passenger volume, survival analysis, travel restriction

## Abstract

From 1 January 2022 to 4 September 2022, a total of 53 996 mpox cases were confirmed globally. Cases are predominantly concentrated in Europe and the Americas, while other regions are also continuously observing imported cases. This study aimed to estimate the potential global risk of mpox importation and consider hypothetical scenarios of travel restrictions by varying passenger volumes (PVs) via airline travel network. PV data for the airline network, and the time of first confirmed mpox case for a total of 1680 airports in 176 countries (and territories) were extracted from publicly available data sources. A survival analysis technique in which the hazard function was a function of effective distance was utilised to estimate the importation risk. The arrival time ranged from 9 to 48 days since the first case was identified in the UK on 6 May 2022. The estimated risk of importation showed that regardless of the geographic region, most locations will have an intensified importation risk by 31 December 2022. Travel restrictions scenarios had a minor impact on the global airline importation risk against mpox, highlighting the importance to enhance local capacities for the identification of mpox and to be prepared to carry out contact tracing and isolation.

## Introduction

From 1 January 2022 to 4 September 2022, a total of 52 996 laboratory-confirmed cases of mpox has been identified globally [1]. The World Health Organization (WHO) declared the Public Health Emergency of International Concern on 23 July 2022. Mpox virus belongs to the Orthopoxvirus genus and has been first detected as a human pathogen in the Zaire (currently known as the Democratic Republic of the Congo, DRC) in 1970. Recently detected endemic regions include; Cameroon, Central African Republic, DRC, Nigeria, Republic of the Congo [2, 3]. Transmission to human occurs through close contact with an infected animal or person or a contaminated material. Animal hosts include a variety of rodents and non-human primates, yet the exact reservoir of mpox is yet to be determined [4, 5]. To present, mpox has not been considered to be contagious prior to symptom onset, while several case reports identifying asymptomatic infection raise concerns for the feasibility of controlling the multi-country outbreak [6]. Moreover, since the mean incubation period is estimated at 8.5 days and can be up to around 21 days, incidental importation can easily occur [5, 7, 8].

The risk of mpox has been continuously debated after the eradication of smallpox [3, 9]. Despite knowledge of smallpox vaccination being effective against mpox, local and global smallpox eradication by 1980 lead to cessation of routine vaccination, and especially cohorts born post smallpox eradication is presumed to have no immunity against mpox [7, 9]. Therefore, increased susceptibility to mpox infection especially among younger generations which were born after smallpox eradication may pose a higher risk to infection. Recently, several smallpox vaccines have become registered as a vaccine to protect against mpox [10]. The first documented outbreak of mpox outside of Africa was in the United States of America in 2003 with exposure from prairie dogs, and only several detected events of importations have occurred globally due to travellers arriving from endemic areas and none of them lead to sustained local transmission [4, 11]. The only human-to-human transmission reported outside of Africa was in the United Kingdom (UK) in 2018 [12].

Considering the global travel network, large European cities had a high risk of importing mpox [13]. The current multi-country outbreak in 2022 was first identified in the UK, and sustained local transmission has been mainly observed in Europe and the Americas [2]. Moreover, the disease has been continuously identified in a majority of global locations. The first case was detected on 6 May 2022 in the UK due to importation from Nigeria [2, 14]. While the index case still remain unclear, intra- and international spread of the disease has been observed with evidence of sustained human to human transmission [14, 15]. As of 4 September, countries which have reported a high cumulative number of cases (>3000) globally were the United States of America (*n* = 19 351), Spain (*n* = 6645), Brazil (*n* = 5197), France (*n* = 4646), Germany (*n* = 3493) and the United Kingdom (*n* = 3413) [1]. Outside of African region, eight deaths have been confirmed to present [1]. A unique characteristic of the current spread of mpox cases is that most cases are concentrated among young men, in the population of men who have sex with men (MSM) [1, 12, 15, 16]. Typically mpox is not considered to be a sexually transmitted infection, while it can be transmitted easily during sexual and intimate contact [17]. While global international travel volume has been largely reduced from 2020–2021, in the present year, the International Air Transport Association expects air passenger volume (PV) to be 69% compared to 2019 (pre-COVID-19 pandemic) [18]. Due to resumptions of human movement via airline transportation, travellers could unintentionally cross the border with mpox infection.

Several mathematical modelling techniques have been developed responding to the rapid dissemination of emerging infectious diseases fuelled by airline travel network [19–24]. Practically travel restrictions have been put in place in several boarders during the COVID-19 pandemic, specifically when it was discovered in Wuhan, China and also when a new variant of concern has been identified (i.e. the Omicron variant in South Africa) [25–27]. A recent study estimated the undetected importation risk against the Omicron variant among low and middle income countries due to limited surveillance capabilities [28]. Quantifications of the impact of travel restriction against the risk of importation has been simulated using airline transportation network arriving from Wuhan, China [29]. Several studies have also quantified the delayed epidemic progression given the stringent travel restriction in Wuhan, China [30, 31].

The present study modified the model previously applied against COVID-19 for the quantification of risk of importation [29]. Our study aimed to quantify the global risk of importation using airline transportation data. Using a hazard-based model and the concept of effective distance based on travel network data, we explored patterns of domestic and international population movement. Our findings on travel patterns could contribute to inform public health interventions, especially for understanding the risk of observing an emerging disease across the border.

## Methods

### Dataset and global airline network

The dates of first onset of mpox case for each location (including country, city and/or name of hospital) were extracted from open-access database developed in response to the multi-country outbreak [32, 33] at 5 September 2022. The data included 175 countries or territories. Two authors, RK and DY, independently checked the validity of data against official announcements from WHO and each governmental report. The extracted case information was then matched with the airport information based on the nearest neighbourhood approach. To construct the airline transportation network, we used the ADS-B exchange data [34]. All flights included in the ADS-B exchange data within a single day of 1 December 2019 (before the COVID-19 pandemic) was extracted. The dataset provides a graph of global travel information consisting of 1724 nodes (corresponds to each airport) and 21 704 edges with edge-weights (corresponds to direct flights between two airports with its PV. The PV on a certain flight was estimated as the reported (maximum) number of seats of the airplane. Then, the PV was multiplied by 0.93 for domestic travel and 0.69 for international travel [18].

### Effective distance

To model the impact of airline network on mpox transmission, an idea of *effective distance*, which was introduced by Brockmann and Helbing [35] and frequently used in previous studies for forecasting the global spread of emerging infectious disease such as SARS, influenza H1N1-2009, MERS and COVID-19 [22, 29], was estimated from the airline network. The effective distance is defined from the minimum distance on the adjacency matrix of the network, incorporating the PV-weighted path length and the degree of each node. In other words, the effective distance is a metric quantifying the network distance between each nodes with the weighted edge being proportional to the PV, irrespective of the physical distance between locations.

The effective distance, *d*_*ij*_, between London Heathrow airport (ICAO code: EGLL) and the *i*th airport in the *j*th country is defined as the minimum length among all possible effective paths. The effective paths from Heathrow airport to the *i*th airport with a sequence of *l* transit airports {*a*_Heathrow_, *a*_1_, …, *a*_*l*−1_, *a*_*i*_} is given by
1
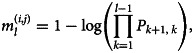
where *P*_*l*,*m*_ denotes the transition probability matrix from the *l*th to the *m*th airport. Each element in *P*_*l*,*m*_ is estimated by 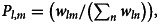 where *w*_*lm*_ is the PV that moved from the *l*th to the *m*th airport. Lastly, *d*_*ij*_ is defined as the minimum effective paths, which is given by
2
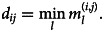


As we will discuss in the next section, since the network structure and its associated effective distance changed after the travel restrictions due to the change in the PV, the effective distance *d*_*ij*_ is dynamically changed in the assumed three scenarios described below.

### Modelling with effective distance: hazard-based approach

We modelled the risk of importing mpox by estimating the survival probability in each country. Let *T* be a random variable indicating the survival time in each airport from the first case in the UK (6 May 2022) to importation at the airport. Also define the survival probability at time of *t* as *F*(*t*) = *P*(*T* > *t*) with the probability density function (pdf) of *f*(*t*) = −*dF*(*t*)/*dt*. *t* indicates day, and *t* = 0 is 6 May 2022. The associated hazard function at time of *t* for importation of mpox from the UK for the *i*th airport in the *j*th country is modelled in the form of Weibull regression model, which is given by
3
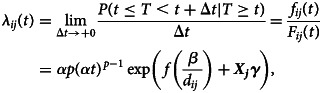
where *p* > 0, *α* > 0, ***γ*** is a vector of regression parameters, *β* is also a regression parameter of interest to measure the impact of the effective distance, *f*() is a penalised smoothing spline function with the degree of freedom of 4, and ***X***_***j***_ is a covariate vector. By using the parameter, *p*, in Equation (3), the Weibull distribution can flexibly model the survival probability even when it is non-convex shape. In addition, other distributions including log-normal and log-logistic distributions were compared and we confirmed that the Weibull distribution can provide the best performance in terms of AIC. This formulation makes the hazard function and the estimated median time of importation be proportional to the effective distance *d*_*ij*_, which is consistent with Shi *et al*. (2021) and Otsuki and Nishiura (2016) [22, 29]. The covariates ***X***_***j***_ include: income per capita at 2022 [36], the proportion of working age at 2020 [37], the proportion of sexual minorities concealing their sexual orientation [38], the proportion of MSM population [39, 40], Socio Demographic Index at 2019 [36] and WHO region (i.e. African Region (AFR), Eastern Mediterranean Region (EMR), European Region (EUR), Region of the Americas (AMR), South-East Asian Region (SEAR), Western Pacific Region (WPR)). Complete case analysis was considered, and thus airports with incomplete covariate information were excluded from the estimation.

The parameters were estimated by the maximum likelihood approach. Then, the future hazard function at 31 December 2022, was predicted by extrapolating the time variable *t*. In order to capture the importation risk from the UK, the following countries currently designated as endemic of mpox is not included in the parameter estimation: Benin, Cameroon, the Central African Republic, the Democratic Republic of the Congo, Gabon, Ghana, Ivory Coast, Liberia, Nigeria, the Republic of the Congo, Sierra Leone and South Sudan [2]. Further, to check the goodness-of-fitting in the model, we calculated the concordance index, which measures the agreement between an observed response and the predictor.

Lastly, we conducted the sensitivity analysis by weakening the assumption in the effective distance: i.e. since the effective distance strongly depends on the assumption that mpox cases has been spreading from the UK, we checked how our result would be changed when weakening this assumption (i.e. not specifying the starting point of the virus spread). More precisely, we used a ‘closeness centrality index’ on the airline network, which measures how attractive a certain airport is in the PV sense, instead of the effective distance in the model. The closeness centrality for the *i*th airport is defined as
4
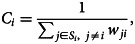
where *w*_*ji*_ is the PV that moved from the *j*th to the *i*th airport and *S*_*i*_ is the set of airports that are connected to the *i*th airport. Then, instead of *d*_*ij*_ in Equation (3), *C*_*i*_ is used. *C*_*i*_ does not assume the starting point of the virus spread, and in that sense we do not make the assumption that the infection is spreading from the UK in this sensitivity model.

### Hypothetical scenarios to estimate relative risk reduction due to travel restriction

To estimate the impact of travel restrictions on the importation risk, we calculated the (observed) cumulative risk at time of *t*, defined by
5
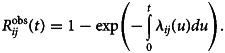


Then we compared 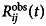 with the cumulative risk of following hypothetical scenarios: (H1) Reduce the current PV from/to infected countries by 50%, (H2) Increase the current PV to ‘pre-COVID-19 level in 2019’ (cf. the current PV was assumed 93% for domestic and 69% for international travel, respectively, compared with the pre-COVID-19 level) [18], and further reduce the PV from/to infected countries by 90%, and (H3) Reduce the current PV to ‘the level at the most severe travel restrictions in 2021’ (i.e. the PV is assumed 61% for domestic and 27% for international travel, respectively, compared with compared with the pre-COVID-19 level), and further reduce the PV from/to infected countries by 50% [18]. Once the regression parameters in Equation (3) was estimated, the cumulative risks based on these scenarios can be calculated by plugging the scenario-specific *d*_*ij*_ into Equation (3): i.e. in a similar way with the calculation of 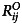, the cumulative risks in (H1)–(H3) are given by
6
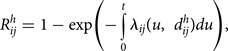

where *h* = 1, 2, 3 indicates scenarios (H1)–(H3), respectively, and 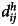 is the effective distance based on each scenario assumption. Lastly, we estimated the relative risk change as follow:
7
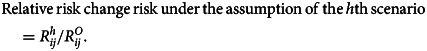


To measures the proportion of expected risk reduction between observed and the hypothetical scenarios the relative risk change was calculated at *t* = 239 (31 December 2022).

## Results

**Fig. 1.**
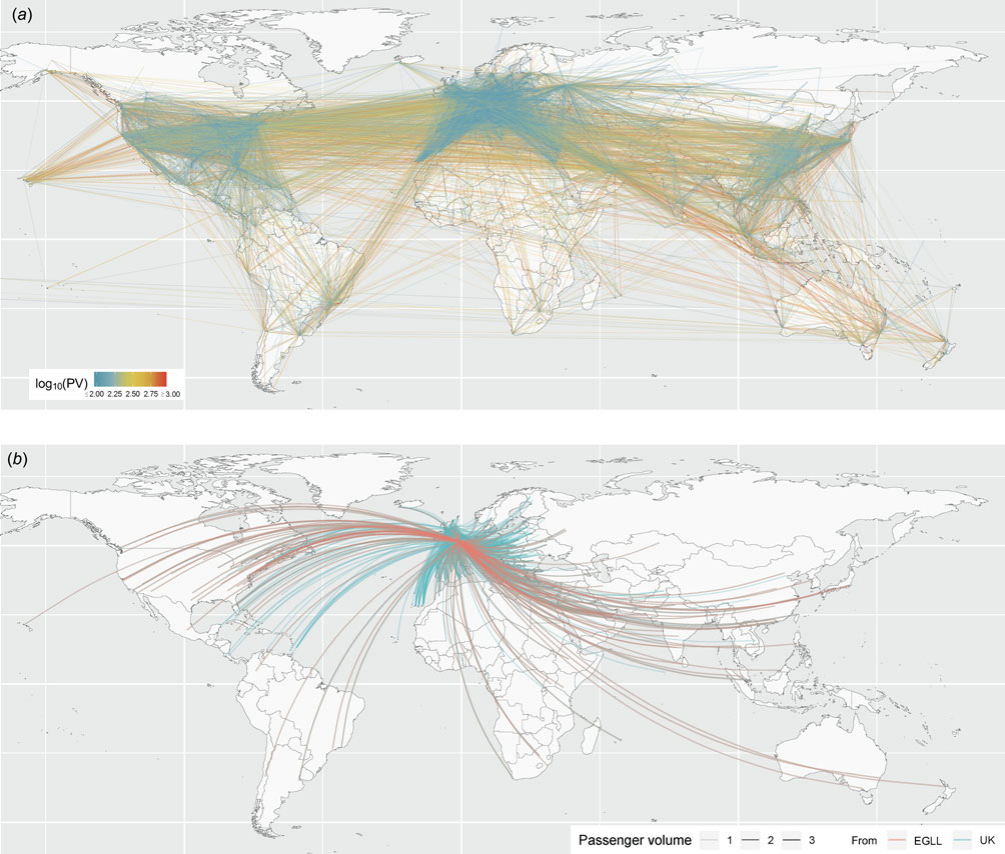
(a) Entire flight network before travel restrictions (as of 1 December 2019). Colour indicates the passenger volume (PV) in log scale. (b) Flight network from the UK and Heathrow Airport (as of 1 December 2019).

Figure 1a shows the entire flight network with the associated PV. PV tends to be higher in intercontinental flights compared to intracontinental flights. Figure 1b indicates PV arriving/departing from the UK and Heathrow Airport. A total of 176 countries (and territories), including the UK, and 1680 airports were included in this analysis. The arrival time ranged from 9 to 48 days since the first case was identified in the UK on 6 May 2022.

**Fig. 2.**
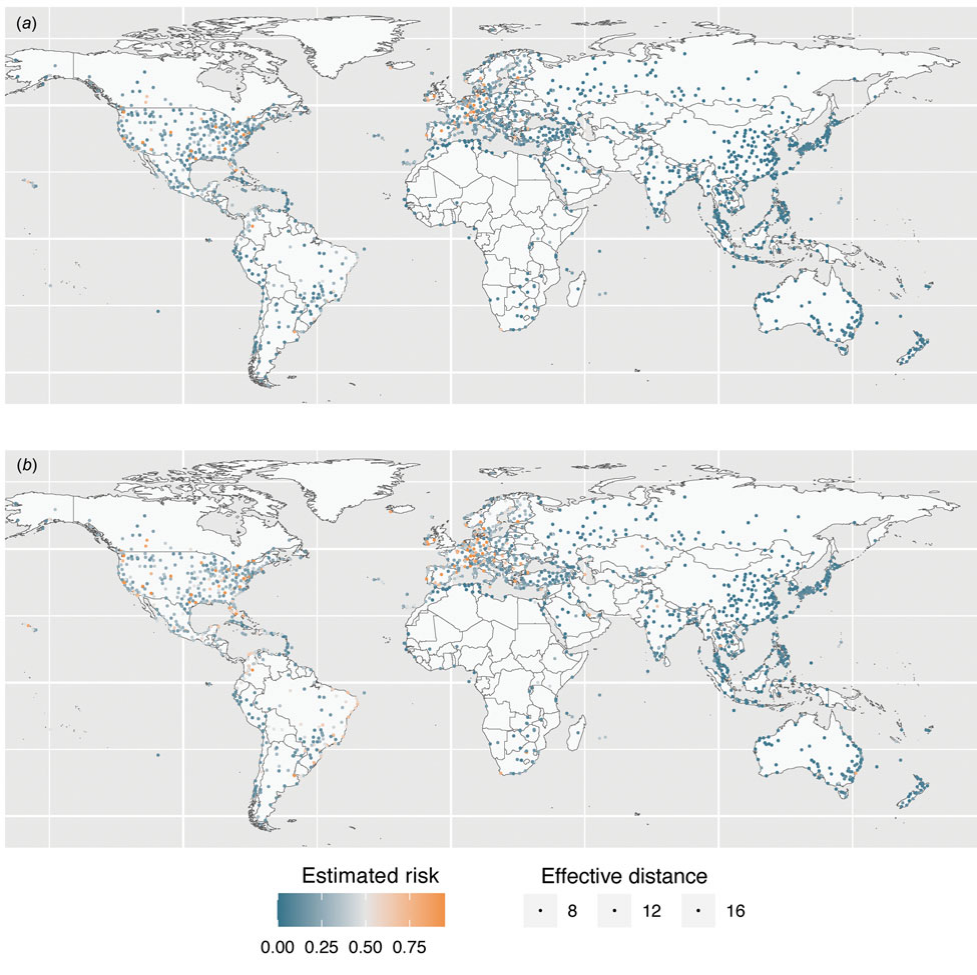
(a) Estimated risk of importation of mpox as of 5 September 2022. Colour indicates the estimated risk and the size of circles indicates the number of inbound passenger volume of each airport. (b) Predicted risk of importation of mpox as of 31 December 2022.

Figures 2a and b show the estimated and predicted cumulative risk 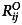 at 5 September and 31 December 2022, respectively. The increased risk over time was similar between WHO regions; the median risk ratios (defined as 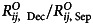) and interquartile range (IQR) in AFR, EMR, EUR, AMR, SEAR, and WPR were 1.56 (IQR = 0.0305), 1.57 (IQR = 0.0593), 1.52 (IQR = 0.0860), 1.50 (IQR = 0.0997), 1.58 (IQR = 0.0196) and 1.58 (IQR = 0.0157), respectively. The estimated concordance index was 0.816, which implicates the model has good fitting ability. To check the robustness of our result, we note that similar results were obtained even in the sensitivity analysis (Supplemental Figure).

**Fig. 3.**
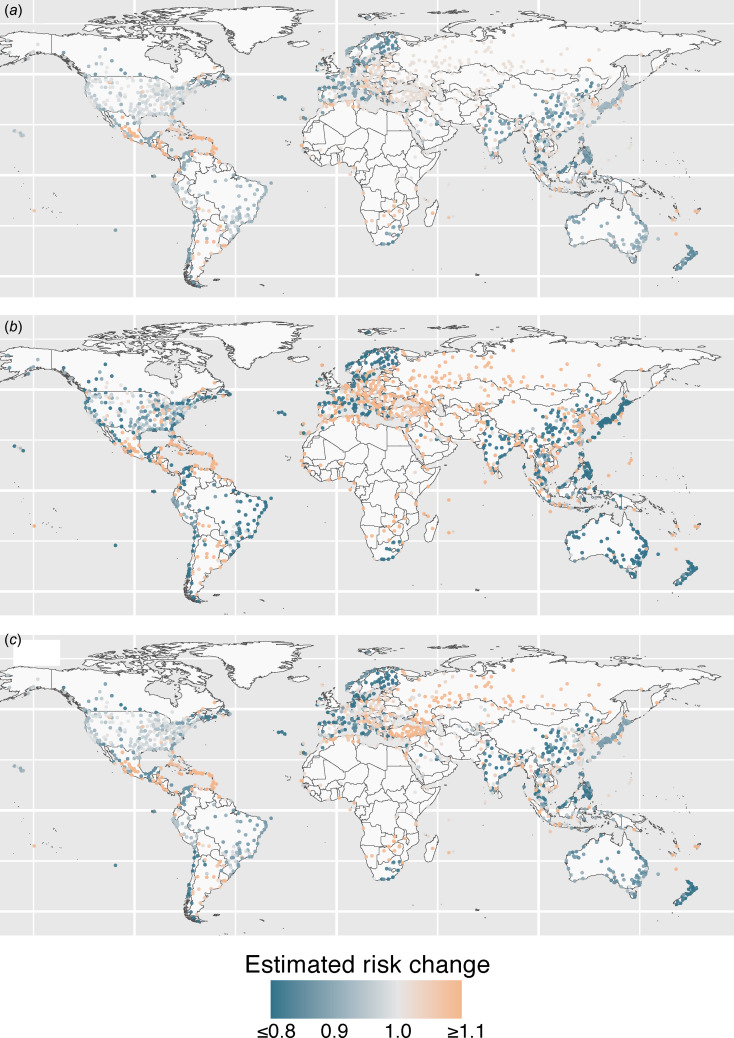
(a) Predicted relative risk change under the assumption of H1 as of 31 December 2022. Colour indicates the estimated relative risk change. (b) Predicted relative risk change under the assumption of H2 as of 31 December 2022. (c) Predicted relative risk change under the assumption of H3 as of 31 December 2022.

Figures 3a–c show the estimated relative risk change under the assumption of (H1)–(H3). The mean (Standard deviation (s.d.), max and min) for the relative risk change were 0.968 (s.d. = 0.075, max = 1.195, min = 0.766), 0.953 (s.d. = 0.240, max = 1.982, min = 0.433) and 0.963 (s.d. = 0.113, max = 1.291, min = 0.685) for (H1)–(H3), respectively. Regarding the geographical distribution of the risk change, in (H1), there was characteristic risk decrease, especially in Europe, Scandinavia, Southeast Asia and Australasia, while there was risk increase in Central America and the Caribbean countries. In (H2), where the overall PV is assumed to return to ‘pre-COVID-19 level’, importation risk increase is observed at similar locations as observed in (H1), while the increase was stronger due to increased travel volume. Surprisingly, areas observing risk decrease was also similar to (H1), and the decrease was stronger even despite increased overall PV. In (H3), risk increase is prevented compared to (H2) due to heavy reduction in the PV assuming 2021 level. However, the strong risk decreases as observed in (H2) is not observed even with significant decrease in overall PV. Interestingly, in all intervention scenarios Central America had an increased risk.

Table 1 shows the estimated risk among top five and bottom five country/territory as of 5 September 2022, 31 December 2022, and three hypothetical travel restriction scenarios. High risks were observed in Switzerland, Luxembourg, Iceland, Qatar, and Malta regardless of estimated time and scenarios. Generally, low risks were observed in Samoa, Palau, Syria, Saint Kitts and Nevis, Christmas Island and Tonga. Apparent changes in the estimated risk reflect the strength of connectedness between London, UK. The estimated risk by scenario did not drastically change in the top or bottom five country/territory. Detailed values by countries/territories are provided in the Supplementary Table.

**Table 1.** Estimated risk among top and bottom five country/territory in Figures 2 and 3

Rank	As of 5 Sep 2022	As of 31 Dec 2022	Scenario 1 (as of 31 Dec 2022)	Scenario 2 (as of 31 Dec 2022)	Scenario 3 (as of 31 Dec 2022)
1	Switzerland (0.930)	Switzerland (0.985)	Switzerland (0.985)	Switzerland (0.987)	Switzerland (0.985)
2	Luxembourg (0.878)	Luxembourg (0.965)	Luxembourg (0.965)	Luxembourg (0.969)	Luxembourg (0.966)
3	Iceland (0.707)	Iceland (0.858)	Iceland (0.859)	Iceland (0.869)	Iceland (0.860)
4	Qatar (0.663)	Qatar (0.823)	Qatar (0.824)	Qatar (0.825)	Qatar (0.824)
5	Malta (0.545)	Malta (0.715)	Malta (0.716)	Malta (0.726)	Malta (0.717)
172	Christmas Island (0.017)	Christmas Island (0.026)	Saint Kitts and Nevis (0.028)	Tonga (0.038)	Saint Kitts and Nevis (0.031)
173	Saint Kitts and Nevis (0.015)	Saint Kitts and Nevis (0.024)	Christmas Island (0.027)	Christmas Island (0.034)	Christmas Island (0.028)
174	Syria (0.013)	Syria (0.020)	Syria (0.021)	Syria (0.024)	Syria (0.021)
175	Palau (0.010)	Palau (0.016)	Palau (0.016)	Palau (0.018)	Palau (0.016)
176	Samoa (0.001)	Samoa (0.001)	Samoa (0.001)	Samoa (0.002)	Samoa (0.001)

The values shown in parenthesis is the estimated risk. Detailed values of 176 countries/territories are provided in the Supplementary Table.

## Discussion

Employing a hazard based model and utilising the idea of effective distance, the present study estimated the importation risk against mpox. Assuming that the current flight volume would be maintained, the risk of importation by 31 December 2022 is expected to be substantial in multiple locations, including areas which yet have experienced sustained local transmission (Fig. 2). Strong risks are seen in locations with large PV with the presence of closely connected flights irrespectively to the actual distance from London, UK. Our result highlights the importance to enhance surveillance against mpox in nations with a high risk of importing mpox.

To confirm a hypothetical scenario where travel restrictions are imposed, we reduced PV from/to countries already detected to have mpox importation. The reduction of PV by 50% may be considered as a situation when travel recommendations were made not to travel in these locations unless necessary (H1 and H3). The reduction of PV by 90% may be considered as a situation when strict travel restrictions were implemented to/from countries identifying mpox (H2). The relative risk reduction given travel restriction only had a minor effect to the risk of importation (Fig. 3a). To verify the sensitivity to different PV on the relative risk reduction, we changed the PV from/to identified location, and the degree of reduction in overall PV considering the travel volume in 2019 (pre-COVID-19 level; high PV) and 2021 (least travel volume during the COVID-19 pandemic; low PV). While minor changes in the risk is observed, varying the global airline PV and travel restrictions from/to identified locations did not strongly contribute to modify the risk of importation (Fig. 3b and c), suggesting that the degree of PV has a nonlinear effect on the risk reduction, and the optimal size of volume reduction may depend on the connectivity between each airport network as also discussed previously [29]. Therefore, our hypothetical scenarios examined that practical implementation of travel restrictions or recommendations to reduce PV in order to minimise the risk of importing mpox cases may not be an efficient strategy. Rather, intensified contact tracing and isolation would be more important once a single case is identified in a new location.

Several limitations must be discussed. First, this study estimated the probability of importation using only airline network data, while sea and ground (automobiles and railway) network are also drivers to human mobility. Second, since we relied on airline transportation data within a single day in 1 December 2019, our analysis could only take into account of the change in the airline travel volume by 2022. Therefore, the changes in network structure due to the COVID-19 pandemic, or by the humanitarian crisis occurring between Ukraine and Russia may not have been adequately addressed, which may result to affect the precision on our estimate. Third, we defined that the spread of mpox originated from London, UK and estimated the global importation risk. However, similar results were obtained in our sensitivity analysis relaxing this assumption. Fourth, our analysis focused on the risk of importation, and therefore the risk of local transmission given importation is not quantified. Despite these limitations, our projection exercise highlights the propagating global risk of importation of mpox cases using airline transportation network.

In conclusion, travel restrictions can impose strong economic and social impact, and thus careful evidence-based decision process is necessary [26]. While our simple model may not fully capture the complex dynamics of global disease transmission, our simulation showed that in the case of mpox, airline travel restrictions may not be the practical intervention to prevent importation in most areas. Instead of preventing the importation of mpox cases via airline networks, countries especially considered to have a high risk of importation should enhance local capacities for the identification of mpox and prepare to carry out contact tracing and isolation.

## Data Availability

The data underlying this article and R programs will be shared on reasonable request to the corresponding author.

## Supplementary material

For supplementary material accompanying this paper visit http://doi.org/10.1017/S0950268823000456. click here to view supplementary material

## References

[1] Multi-country outbreak of monkeypox (2022) https://www.who.int/publications/m/item/multi-country-outbreak-of-monkeypox--external-situation-report--5---7-september-2022 (accessed 17 October 2022).

[2] Multi-country monkeypox outbreak: situation update (2022) https://www.who.int/emergencies/disease-outbreak-news/item/2022-DON392 (accessed 17 October 2022).

[3] Bunge EM (2022) The changing epidemiology of human monkeypox—A potential threat? A systematic review. PLoS Neglected Tropical Diseases 16, e0010141–e0010141. 35148313 10.1371/journal.pntd.0010141 PMC8870502

[4] Monkeypox (2022) https://www.who.int/news-room/fact-sheets/detail/monkeypox (accessed 17 October 2022).

[5] McCollum AM and Damon IK (2014) Human monkeypox. Clinical Infectious Diseases 58, 260–267. 24158414 10.1093/cid/cit703 PMC5895105

[6] De Baetselier I (2022) Retrospective detection of asymptomatic monkeypox virus infections among male sexual health clinic attendees in Belgium. Nature Medicine 11, 2288–2292. 10.1038/s41591-022-02004-w PMC9671802 35961373

[7] Grant R, Nguyen L-BL and Breban R (2020) Modelling human-to-human transmission of monkeypox. Bulletin of the World Health Organization 98, 638–640. 33012864 10.2471/BLT.19.242347 PMC7463189

[8] Miura F (2022) Estimated incubation period for monkeypox cases confirmed in the Netherlands, May 2022. Eurosurveillance 27, 2200448. 35713026 10.2807/1560-7917.ES.2022.27.24.2200448 PMC9205160

[9] Breman JG and Henderson DA (1998) Poxvirus dilemmas—monkeypox, smallpox, and biologic terrorism. Mass Medical Soc 339, 556–559. 10.1056/NEJM199808203390811 9709051

[10] Gruber MF (2022) Current status of monkeypox vaccines. npj Vaccines 7, 1–3. 35977979 10.1038/s41541-022-00527-4 PMC9385639

[11] Centers for Disease C, Prevention (2003) Update: multistate outbreak of monkeypox--Illinois, Indiana, Kansas, Missouri, Ohio, and Wisconsin, 2003. MMWR Morbidity and Mortality Weekly Report 52, 642–646. 12855947

[12] Vaughan A (2020) Human-to-human transmission of monkeypox virus, United Kingdom, October 2018. Emerging Infectious Diseases 26, 782–785. 32023204 10.3201/eid2604.191164 PMC7101111

[13] Au NH (2022) Potential for monkeypox exportation from west and Central Africa through global travel networks. Journal of Travel Medicine 29, taac072. 35642580 10.1093/jtm/taac072

[14] Vivancos R (2022) Community transmission of monkeypox in the United Kingdom, April to May 2022. Eurosurveillance 27, 2200422. 35656834 10.2807/1560-7917.ES.2022.27.22.2200422 PMC9164677

[15] Perez Duque M (2022) Ongoing monkeypox virus outbreak, Portugal, 29 April to 23 May 2022. Eurosurveillance 27, 2200424. 35656830 10.2807/1560-7917.ES.2022.27.22.2200424 PMC9164676

[16] Ferraro F (2022) Letter to the editor: multiple introductions of MPX in Italy from different geographic areas. Eurosurveillance 27, 2200456. 35686566 10.2807/1560-7917.ES.2022.27.23.2200456 PMC9198655

[17] Guarner J, del Rio C and Malani PN (2022) Monkeypox in 2022—what clinicians need to know. JAMA 328, 139–140. 35696257 10.1001/jama.2022.10802

[18] Air Passenger Numbers to Recover in 2024 (2022) https://www.iata.org/en/pressroom/2022-releases/2022-03-01-01/ (accessed 17 October 2022).

[19] Colizza V (2006) The role of the airline transportation network in the prediction and predictability of global epidemics. Proceedings of the National Academy of Sciences of the United States of America 103, 2015–2020. 16461461 10.1073/pnas.0510525103 PMC1413717

[20] Khan K (2009) Spread of a novel influenza A (H1N1) virus via global airline transportation. New England Journal of Medicine 361, 212–214. 19564630 10.1056/NEJMc0904559

[21] Poletto C (2014) Assessing the impact of travel restrictions on international spread of the 2014 West African Ebola epidemic. Eurosurveillance 19, 20936. 25358040 10.2807/1560-7917.es2014.19.42.20936 PMC4415609

[22] Otsuki S and Nishiura H (2016) Reduced risk of importing ebola virus disease because of travel restrictions in 2014: a retrospective epidemiological modeling study. PLoS One 11, e0163418–e0163418. 27657544 10.1371/journal.pone.0163418 PMC5033593

[23] Nah K (2016) Predicting the international spread of Middle East respiratory syndrome (MERS). BMC Infectious Diseases 16, 356–356. 27449387 10.1186/s12879-016-1675-z PMC4957429

[24] Tatem AJ, Rogers DJ and Hay SI (2006) Global transport networks and infectious disease spread. Advances in Parasitology 62, 293–343. 16647974 10.1016/S0065-308X(05)62009-X PMC3145127

[25] Grépin KA (2021) Evidence of the effectiveness of travel-related measures during the early phase of the COVID-19 pandemic: a rapid systematic review. BMJ Global Health 6, e004537–e004537. 10.1136/bmjgh-2020-004537 PMC7969755 33722793

[26] Meier BM (2022) Travel restrictions and variants of concern: global health laws need to reflect evidence. Bulletin of the World Health Organization 100, 178–178A. 35261400 10.2471/BLT.21.287735 PMC8886257

[27] Bai Y (2022) International risk of SARS-CoV-2 omicron variant importations originating in South Africa. Journal of Travel Medicine 29, taac073. 35704033 10.1093/jtm/taac073 PMC9278166

[28] Bi K (2023) The risk of SARS-CoV-2 Omicron variant emergence in low and middle-income countries (LMICs). Epidemics 42, 100660. 36527867 10.1016/j.epidem.2022.100660 PMC9727964

[29] Shi S (2020) Travel restrictions and SARS-CoV-2 transmission: an effective distance approach to estimate impact. Bulletin of the World Health Organization 98, 518–529. 32773897 10.2471/BLT.20.255679 PMC7411317

[30] Chinazzi M (2020) The effect of travel restrictions on the spread of the 2019 novel coronavirus (COVID-19) outbreak. Science (New York, NY) 368, 395–400. 10.1126/science.aba9757 PMC7164386 32144116

[31] Anzai A (2020) Assessing the impact of reduced travel on exportation dynamics of novel coronavirus infection (COVID-19). Journal of Clinical Medicine 9, 601. 32102279 10.3390/jcm9020601 PMC7073579

[32] Kraemer MUG (2022) Tracking the 2022 monkeypox outbreak with epidemiological data in real-time. The Lancet Infectious Diseases 22, 941–942. 35690074 10.1016/S1473-3099(22)00359-0 PMC9629664

[33] Global.health Monkeypox (accessed 2022-09-05).

[34] Exchange ADS-B. Available at https://www.adsbexchange.com/data/ (accessed 17 October 2022).

[35] Dirk B and Dirk H (2013) The hidden geometry of complex, network-driven contagion phenomena. Science (New York, N.Y.) 342, 1337–1342. 24337289 10.1126/science.1245200

[36] Gross Domestic Product Per Capita 1960-2050. Available at https://ghdx.healthdata.org/record/ihme-data/global-gdp-per-capita-1960-2050 (accessed 17 October 2022).

[37] Population ages 15-64 (% of total population). Available at https://data.worldbank.org/indicator/SP.POP.1564.TO.ZS?name_desc=false (accessed 17 October 2022).

[38] Pachankis JE and Bränström R (2019) How many sexual minorities are hidden? Projecting the size of the global closet with implications for policy and public health. PLoS One 14, e0218084–e0218084. 31194801 10.1371/journal.pone.0218084 PMC6564426

[39] The Key Populations Atlas. Available at https://kpatlas.unaids.org/ (accessed 17 October 2022).

[40] Recommended population size estimates of men who have sex with men. Available at https://www.who.int/publications/i/item/9789240015357 (accessed 17 October 2022).

