# Supplementary figures and images for: Impact of airline network on the global importation risk of mpox, 2022
Source: Epidemiol Infect. 2023 Mar 21;151:e60. doi: 10.1017/S0950268823000456 (PMC10126888; doi:10.1017/S0950268823000456)

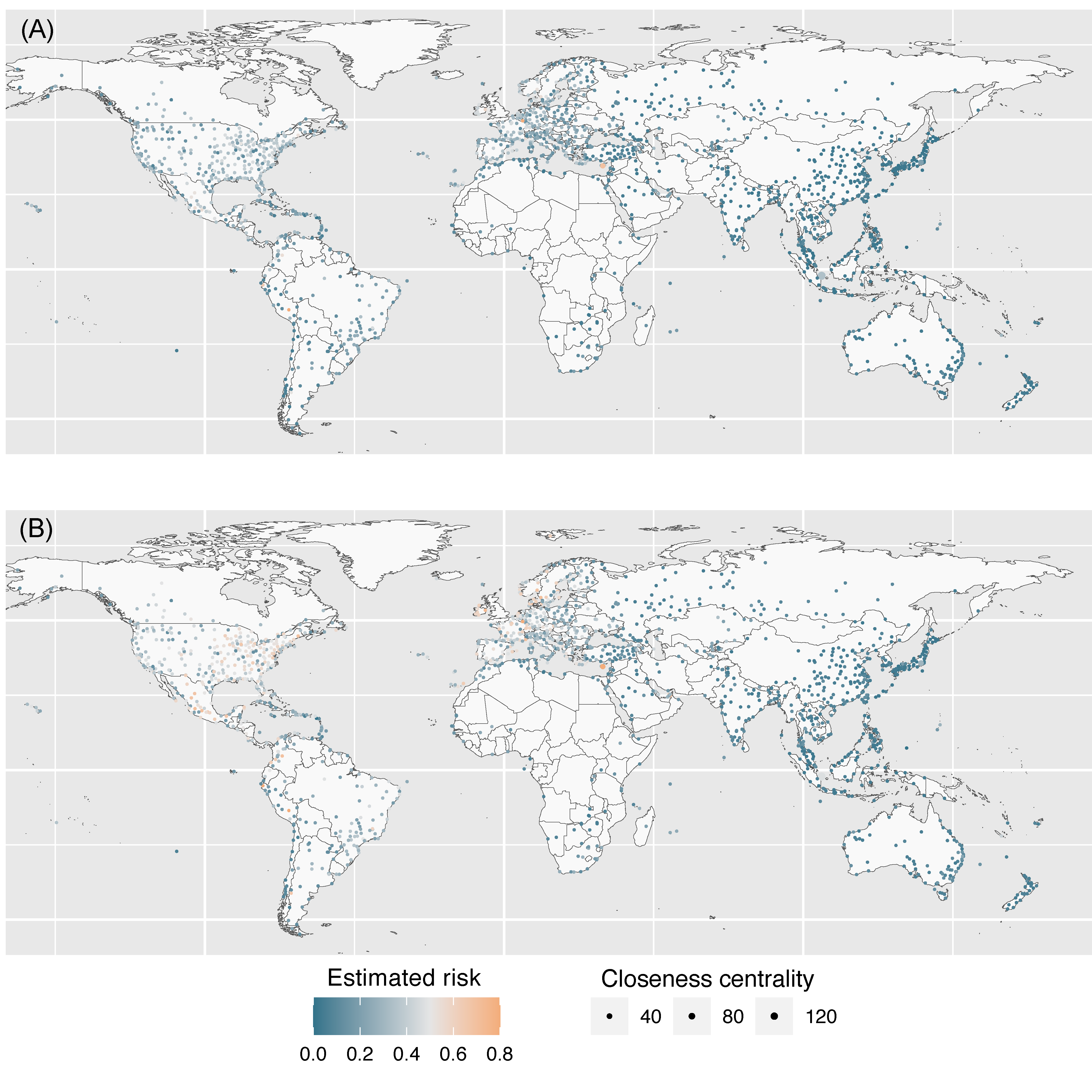

Supplement: Supplementary file 1 [file hygsup.zip › S0950268823000456sup001.tif]

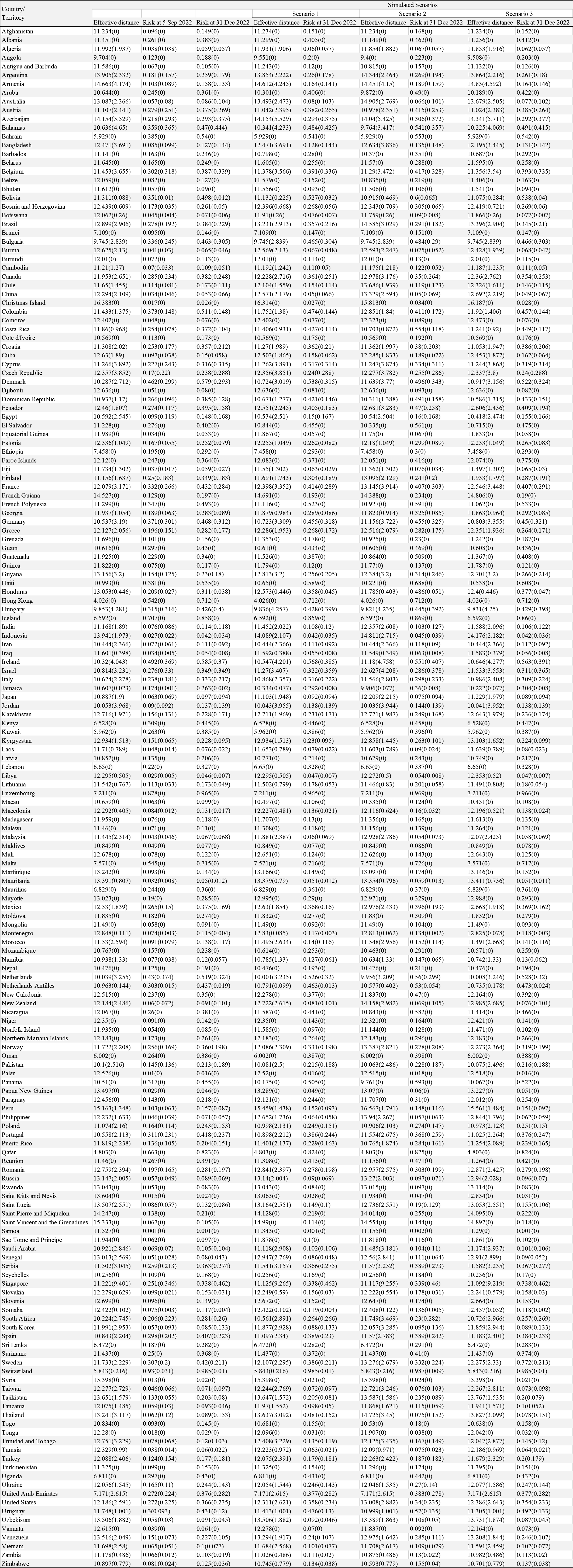

Supplement: Supplementary file 1 [file hygsup.zip › S0950268823000456sup002.tif]
